# Rapid sequence intubation: a survey of current practice in the South African pre-hospital setting

**DOI:** 10.1186/s12245-021-00368-3

**Published:** 2021-08-17

**Authors:** Johanna Catharina Botha, Andrit Lourens, Willem Stassen

**Affiliations:** 1 Division of Emergency Medicine, Faculty of Health Sciences, University of Cape Town, Cape Town, South Africa; 2 School of Nursing, Midwifery and Health, Faculty of Health and Life Sciences, Coventry University, Coventry, UK

**Keywords:** Rapid sequence intubation (RSI), Pre-hospital, Minimum standards, Education and training, System requirements, Comprehensive clinical governance, South Africa

## Abstract

**Background:**

Rapid sequence intubation (RSI) is an advanced airway skill commonly performed in the pre-hospital setting globally. In South Africa, pre-hospital RSI was first approved for non-physician providers by the Health Professions Council of South Africa in 2009 and introduced as part of the scope of practice of degree qualified Emergency Care Practitioners (ECPs) only. The research study aimed to investigate and describe, based on the components of the minimum standards of pre-hospital RSI in South Africa, specific areas of interest related to current pre-hospital RSI practice.

**Methods:**

An online descriptive cross-sectional survey was conducted amongst operational ECPs in the pre-hospital setting of South Africa, using convenience and snowball sampling strategies.

**Results:**

A total of 87 participants agreed to partake. Eleven (12.6%) incomplete survey responses were excluded while 76 (87.4%) were included in the data analysis. The survey response rate could not be calculated. Most participants were operational in Gauteng (*n* = 27, 35.5%) and the Western Cape (*n* = 25, 32.9%). Overall participants reported that their education and training were perceived as being of good quality. The majority of participants (*n* = 69, 90.8%) did not participate in an internship programme before commencing duties as an independent practitioner. Most RSI and post-intubation equipment were reported to be available; however, our results found that introducer stylets and/or bougies and end-tidal carbon dioxide devices are not available to some participants. Only 50 (65.8%) participants reported the existence of a clinical governance system within their organisation. Furthermore, our results indicate a lack of clinical feedback, deficiency of an RSI database, infrequent clinical review meetings and a shortage of formal consultation frameworks.

**Conclusion:**

The practice of safe and effective pre-hospital RSI, performed by non-physician providers or ECPs, relies on comprehensive implementation and adherence to all the components of the minimum standards. Although there is largely an apparent alignment with the minimum standards, recurrent revision of practice needs to occur to ensure alignment with recommendations. Additionally, some areas may benefit from further research to improve current practice.

**Supplementary Information:**

The online version contains supplementary material available at 10.1186/s12245-021-00368-3.

## Background

Rapid sequence intubation (RSI) is an advanced airway skill commonly performed in the pre-hospital setting globally, by physician or non-physician providers [1, 2]. RSI is regarded the gold standard for advanced airway management in critically ill and/or injured patients, mainly due to the optimal conditions created to facilitate endotracheal intubation (ETI) and by restricting the physiological effects of the procedure [2, 3]. Arguably, the pre-hospital environment is not the ideal setting to perform high-risk procedures, such as RSI; however, some research suggests that certain patient groups, like those with severe traumatic brain injuries, may require immediate advanced airway interventions [4, 5].

Worldwide, experts raised concerns about the safety, efficacy, harm and delays that non-physician pre-hospital RSI may cause; nevertheless, the heterogeneity of available research makes comparisons and generalisability of conclusions regarding the value problematic [6, 7]. More recent studies, however, indicate higher endotracheal tube (ETT) first-pass and overall success rates amongst paramedics and/or student paramedics compared to earlier research [8–11]. Moreover, newer literature suggests that highly trained non-physician providers and increased experience may improve ETT pass success rates and reduce adverse events [12]. Lastly, the important role of a well-established clinical governance system, to deliver safe, quality patient care before, during and after ETI, ensuring optimal oxygenation, ventilation, normocapnia and normovolemia as well as preventing aspiration and other adverse effects associated with increased mortality and morbidity, should be underscored [13–15].

In South Africa (SA), pre-hospital RSI was first approved to be performed by degree qualified Emergency Care Practitioners (ECPs), non-physicians, by the Health Professions Council of South Africa (HPCSA) in 2009 [16]. With this addition to the scope of practice of ECPs, the HPCSA laid down the minimum standards forming the foundation and supportive framework to guide the implementation process and to ensure safe and effective practice [16]. Furthermore, a Position Statement published in 2010, endorsed by the Emergency Medicine Society of South Africa (EMSSA) and the Resuscitation Council of Southern Africa, provided additional details regarding RSI practice [1]. Since the approval, it is not known to what extent the minimum standards filtered down to the end-user level to support and enable ECPs to perform RSI safely and effectively.

Being a high-risk skill, RSI involves much more than merely passing the ETT [17]; therefore, to facilitate pre-hospital RSI, specific requirements such as training, system requirements and an appropriate clinical governance framework need to be considered before implementation and practice [1]. RSI, especially performed by non-physician providers, is a heavily debated topic globally [5, 7]. As the concerns with regard to the safety and effectiveness are universal, a description of current pre-hospital non-physician RSI practice, albeit of an upper-middle-income country, may have a significant impact on the practice of the skill by non-physicians globally.

The research study aimed to investigate and describe, based on the components of the minimum standards of pre-hospital RSI in SA, specific areas of interest related to current pre-hospital RSI practice. The specific objectives were to describe pre-hospital training, system requirements and clinical governance systems in SA.

## Methods

### Study design

A descriptive cross-sectional survey was conducted amongst operational ECPs in the South African pre-hospital setting.

### Study setting and population

SA is an upper-middle-income country, in Southern Africa, with a population of approximately 58 million in 2019 [18]. Pre-hospital emergency care (PEC) is provided by public, private or non-governmental organisations and includes platforms, such as ground ambulances, response vehicles, specialised ambulances and aeromedical.

Eligible study participants were operational ECPs, registered with the HPCSA, working in the South African pre-hospital setting. As mentioned, ECPs are the only group of emergency care providers allowed to perform pre-hospital RSI. With the implementation of the new emergency care clinical practice guidelines published in 2018, medication-facilitated intubation is no longer allowed [19]. For the study, the term “operational” entailed any PEC duties performed, in the capacity of an ECP, whether it is full-time or part-time with or without compensation.

Non-probability, convenience and snowball sampling strategies were used; however, due to the lack of reliable or published information on the exact number of operational ECPs in the South African pre-hospital setting, a sample size could not be calculated. Many HPCSA-registered ECPs are currently working abroad or employed in non-operational positions [20].

### Data collection

We collected our data, using a trustworthy online survey tool, SurveyMonkey®. The survey (Additional file 1) was mindfully designed, using a variety of reputable resources [21–23]. To establish face validity, individuals knowledgeable on the study content and questionnaire design evaluated the survey and offered recommendations after which a pilot study was conducted amongst experienced, registered HPCSA ECPs working abroad. Recommendations were incorporated and the survey refined.

An electronic invitation and infographic, containing information and access to the online survey, were distributed to potential participants and key role players across SA, who in turn further distributed the survey invitation to other potential participants. Data were collected between 18 July and 18 September 2019. Electronic reminders were used to enhance study participation.

### Data analysis

Data were analysed using IBM SPSS Statistics for Windows, version 25 [24]. Shapiro-Wilk tests were conducted to assess for normality. Demographic information (frequency, percentage, median (M), interquartile range (IQR) and range) and survey responses (frequency and percentage) were expressed using descriptive statistics and presented with tables and graphs. The Pearson's chi-square test of independence was used to determine relationships between categorical variables and the Phi correlation coefficient to determine the strength of association. A *p*-value of < 0.05 was considered statistically significant.

## Results

### Demographics

Eighty-seven participants agreed to partake and 11 (12.6%) incomplete responses were excluded while the remainder (*n* = 76, 87.4%) were included in the data analysis. Though the survey completion rate was 87%, due to the sampling technique, the response rate could not be calculated as the exact sample population could not be determined (denominator unknown).

Most participants were operational in Gauteng (*n* = 27, 35.5%) and the Western Cape (*n* = 25, 32.9%) (Table 1). The overall years of emergency medical service (EMS) experience ranged between < 1 and 27 (M = 7, IQR = 5–13) years while the years of experience as an ECP ranged between < 1 and 12 (M = 3, IQR = 2–6) years.

**Table 1:** Participant characteristics

Characteristics	*n* (%)
***Province***	
Gauteng	27 (35.5%)
Western Cape	25 (32.9%)
KwaZulu-Natal	13 (17.1%)
Eastern Cape	5 (6.6%)
Free State	2 (2.6%)
Mpumalanga	1 (1.3%)
Northern Cape	1 (1.3%)
Limpopo	1 (1.3%)
North West	1 (1.3%)
	**76 (100%)**
***Job title***	
Operational paramedic (ECP^*a*^)	47 (61.8%)
Lecturer/Instructor/Trainer in EMC^*b*^/EM^*c*^	17 (22.4%)
Operational flight paramedic (ECP^*a*^)	7 (9.2%)
Manager/Administrative/Researcher in EMC^*b*^/EM^*c*^	4 (5.3%)
No response	1 (1.3%)
	**76 (100%)**
***Years of total experience in pre-hospital EMS***^***d***^***systems***	
0–10 years	49 (64.5%)
11–20 years	21 (27.6%)
21–30 years	6 (7.9%)
	**76 (100%)**
***Years of experience as ECP***^***a***^	
0–5 years	56 (73.7%)
6–12 years	20 (26.3%)
	**76 (100%)**
***Organisation***	
Private	43 (56.6%)
Public (Government)	23 (30.3%)
University/Training Institution	6 (7.9%)
Non-Governmental Organisation (NGO)	4 (5.3%)
	**76 (100%)**

^a^Emergency Care Practitioner (ECP), ^b^Emergency Medical Care (EMC), ^c^Emergency Medicine (EM), ^d^Emergency Medical Services (EMS)

### Training

**Fig. 1:**
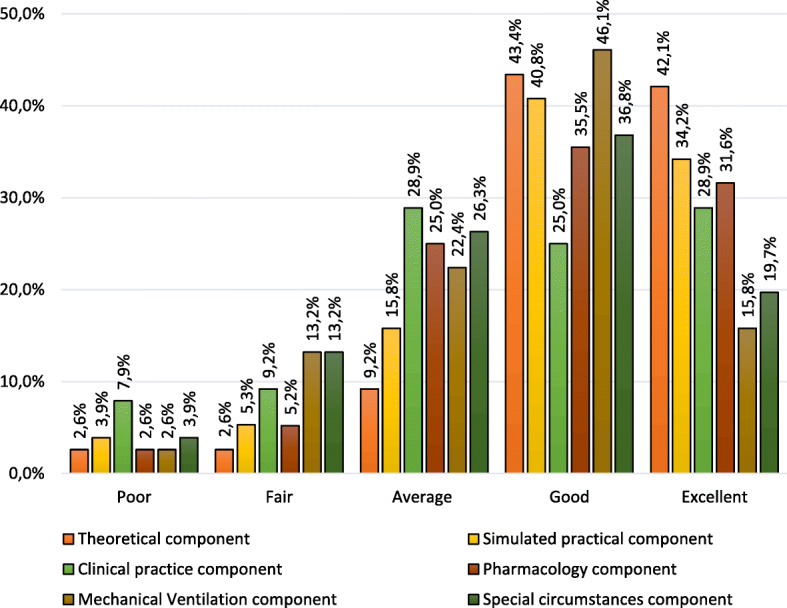
Perceived quality of RSI training received during tertiary education

For the theoretical (43.4%), simulated practical (40.8%), pharmacology (35.5%), mechanical ventilation (46.1%) and special circumstances (36.8%) components, most participants reported the quality of education and training received to be good (Fig. 1). The quality of the clinical practice component was scored either average or excellent (28.9% each) by most while 25% reported the quality to be good. The theoretical component was perceived to be of the highest quality (94.7% selected average, good or excellent) followed by pharmacology and simulated practice, while special circumstances and clinical practice were reported to be of lower quality (Additional file 2: Table S1).

Most participants (*n* = 69, 90.8%) did not partake in an internship programme (e.g. working with a qualified ECP for a period after qualification) before commencing independent duties (Table 2). Almost 60% of participants (*n* = 45, 59.2%) have not participated in formal education and training activities specifically focusing on RSI, since graduation. No association was found between years’ experience as an ECP (≤5 years or 6–12 years) and participation in an internship programme (*p* = 0.877) as well as between years’ experience as an ECP (≤5 years or 6–12 years) and participation in formal RSI activities since graduation (*p* = 0.132).

**Table 2:** Participation in an internship programme after qualification as an ECP

Internship period	*n* (%)
No participation	69 (90.8%)
< 1 month	4 (5.3%)
1–3 months	2 (2.6%)
> 3 months	1 (1.3%)
	**76 (100%)**

### System requirements

**Fig. 2:**
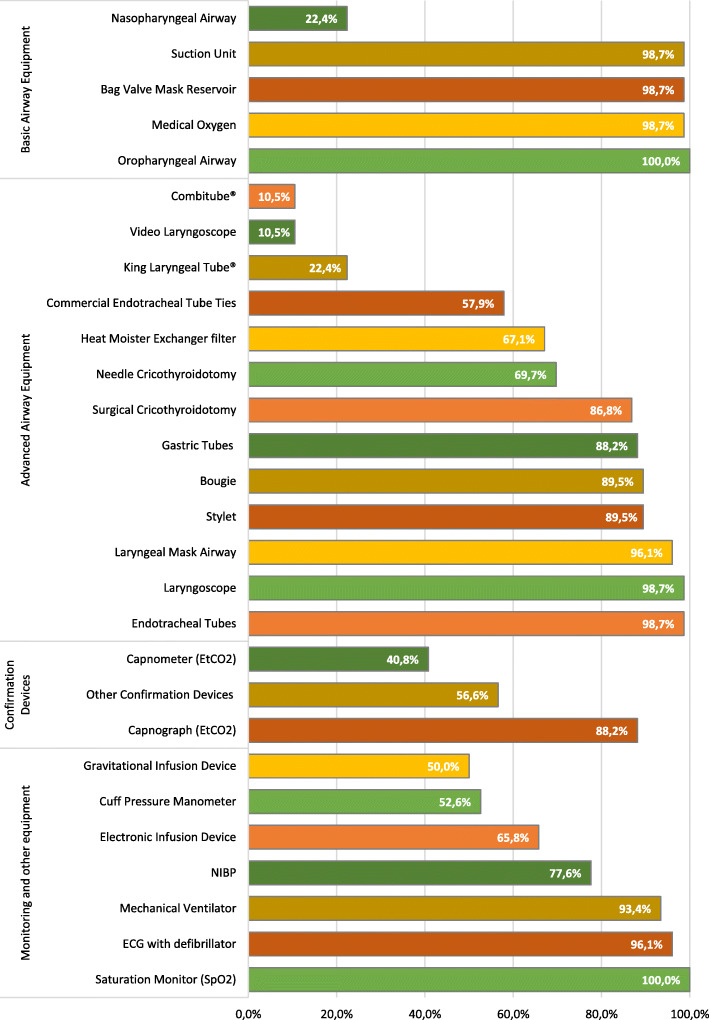
Equipment available to perform RSI and post-intubation management

The availability of equipment to perform RSI and post-intubation management is indicated in Fig. 2. All participants (*n* = 76, 100%) reported having an alternative airway device available, although the type of device varied. Sixty-eight (89%) participants have either a bougie or a stylet available, 63 (82.9%) reported the availability of both and three (4%) neither device. End-tidal carbon dioxide (EtCO_2_) measurement and monitoring capability were reported as 31 (40.8%) having a capnometer and 67 (88.1%) a capnograph whereas five participants reported not having any EtCO_2_ device available.

Forty-one (53.9%) participants reported no equipment sharing amongst ECPs, while 32 (42.1%) reported some sharing (missing data for 3 participants) (Additional file 2, Tables S2 and S3). Forty (52.6%) participants reported the availability of an ETT cuff pressure manometer and 8 (10.5%) indicated the availability of a video laryngoscope (VL) while 4 (50%) reported that a VL was shared or is kept at the base. Table 3 depicts VL and ETT cuff manometer availability by EMS organisations. Ketamine, midazolam and morphine (99% each) were more regularly available compared to etomidate (92%) and fentanyl (8%). Similarly, rocuronium (97%) and suxamethonium (92%) were more available, compared to vecuronium (9%).

**Table 3:** Availability of video laryngoscope and ETT cuff manometer per EMS organisation type

Type of EMS organisation	Video laryngoscope *n* (%)	ETT cuff manometer *n* (%)
Public (Government)	2 (2.6%)	5 (6.6%)
Private	3 (3.9%)	27 (35.6%)
Non-Governmental Organisations (NGOs)	3 (3.9%)	4 (5.3%)
University/Training Institution	0 (0%)	4 (5.3%)
	**8 (10.5%)**	**40 (52.6%)**

**Fig. 3:**
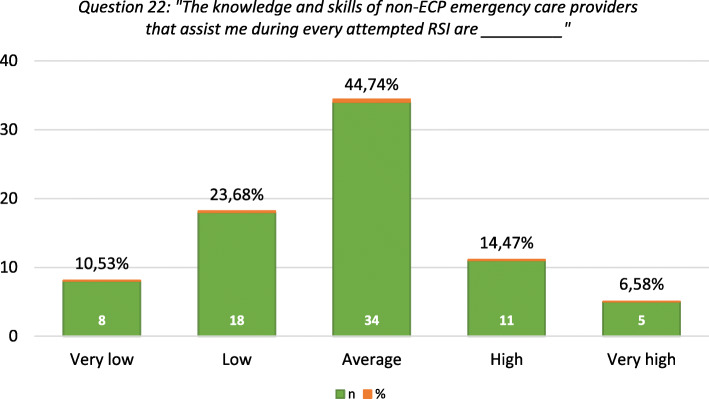
ECP’s perceived level of knowledge and skill of non-ECPs that assist during RSI

Thirty-four (44.7%) participants specified that they have at least one dedicated assistant available during an RSI attempt, 29 (38.2%) at least 2 and 10 (13.2%) at least 3 while 3 (3.9%) have no assistants available. The qualification held by the available RSI assistants is summarised in Additional file 2, Table S4. Most (*n* = 34, 44.74%) participants reported the perceived level of knowledge and skills of non-ECP emergency care providers to assist them during attempted RSI as average (Fig. 3). A few participants (*n* = 5) named the availability of three airway management short courses (namely Evidence-Based Management of Oxygenation, Ventilation and Airway (EMOVA), Airway Interventions & Management in Emergencies and Advanced Airway & Ventilation) in SA. Most participants reported that non-ECPs acquired the necessary knowledge and skills to assist during RSI, by working with an ECP (*n* = 60, 78.9%) and/or through informal training by an ECP (*n* = 8, 10.5%) (Additional file 2, Table S5).

### Comprehensive clinical governance system

Fifty (65.8%) participants reported a continuous quality improvement (CQI) or quality assurance (QA) department or representative within their organisation. Of these, 11 (22%) never, 28 (56%) sometimes and 11 (22%) always receive feedback regarding attempted or performed RSI. Some participants describe the CQI in terms of RSI as “positive”, “constructive”, “discussions taking place”, “problem identification” and “restorative”. In some instances, peer reviews of clinical documentation are performed. Individuals that hold responsibilities for CQI are described as “Doctor”, “EM Physician”, “Senior ECP” and “person from outside the organisation”. To a lesser degree, the CQI process was described as “investigative in nature” with “no continuous training and quality”. In some cases, the person responsible for CQI holds a “lower qualification” compared to an ECP and thus “was not formally taught to perform RSI”.

The submission of clinical documentation for an attempted/performed RSI is always required of 62 (81.6%) participants, sometimes of 4 (5.3%) and never of 10 (13.2%). Most (*n* = 50, 65.8%) participants indicated that clinical review and/or mortality and morbidity meetings to discuss attempted/performed RSI cases occur within the organisation; however, the frequency varied (Table 4). Thirty-one (40.8%) participants reported that all RSI cases are captured on an organisational database, while the remaining was unsure (*n* = 24, 31.6%) or reported no RSI database (*n* = 21, 27.6%). A moderate association was found between organisations with a CQI/QA programme and organisations with an RSI database (*p* = 0.006, *ϕ* 0.316), suggesting those with a CQI/QA programme were more likely to have an RSI database.

**Table 4:** Clinical review and/or mortality and morbidity meetings

Number of meetings	*n* (%)
Never (zero per year)	26 (34.2%)
Rarely (once a year)	19 (25.0%)
Sometimes (every 6 months)	11 (14.5%)
Usually (every 3 months)	7 (9.2%)
Always (every month)	12 (15.8%)
Missing data	1 (1.3%)
	**76 (100%)**

Fifty-two (68.4%) participants reported a formal consultation framework (e.g. senior ECP, peer and/or physician) within their organisation while 60 (78.9%) occasionally consult informally with regard to performing RSI. A strong association was found between organisations with a formal consultation framework and those with a CQI/QA programme (*p* < 0.001, *ϕ* 0.583) but not between the presence of a CQI/QA programme and practitioners who occasionally consult informally (*p* = 0.134). Organisations with a formal consultation framework were more likely to also have a CQI/QA programme.

## Discussion

To our knowledge, this study is the first to investigate and describe, based on the components of the minimum standards, specific areas related to South African pre-hospital RSI practice; therefore, the findings will be valuable to understand current practice and make recommendations for improvement and further research.

### Demographics

Considering the relatively recent addition of RSI to the scope of practice for ECPs [16] and the phased introduction of degree programmes and professionalisation of PEC education and training in SA since the early 2000s [25, 26], a less clinically experienced ECP workforce, as we found, may be expected. Moreover, career progression and the international market may further compound the limited clinical experience of the workforce [20]. In the context of safe and effective pre-hospital RSI, this may be a concern, as the literature suggests an association between increased advanced airway management and RSI experience and higher ETI success rates and less adverse events contributing to lower mortality and morbidity [27–29]. The provincial representation of participants coincides with the highest populated provinces accumulatively, with the top three ranking provinces accounting for roughly half the country’s population [30], and corresponds with the known distribution of operational ECPs and location of tertiary education institutions delivering ECP level education [25, 26].

### Training

#### Tertiary education

Though the overall perceived quality of RSI tertiary education and training components, especially the theoretical component, were found to be commendable, the clinical practice and special circumstances components were suggested to be of lower quality. Various factors could be contributory including the method of delivery and/or inadequate learning opportunities during clinical practice placements. Recent literature suggests that the current education and training methods in SA do not seem to meet clinical practice learning objectives, which may lead to the potential inadequate preparation of paramedics for post-qualification independent practice [31]. These components may require enhancement through extensive collaboration between the relevant stakeholders to improve students’ learning experiences [27, 31].

A limited number of opportunities to perform supervised RSI as a student ECP may be an additional factor potentially impacting the safety and quality of RSI by newly qualified practitioners as complication rates improve only after about 30 procedures [28]. Pre-hospital RSI experts advise that irrespective of being a physician or non-physician, ETI proficiency or competency requires considerably higher numbers to achieve at least a 90% success rate [32, 33]. ECP students require 35 (11 pre-hospital) supervised ETIs and/or RSIs during their studies, 24 performed in an operating room under the supervision of an anaesthesiologist [11], which are contextually very different from pre-hospital RSI. This low frequency of supervised pre-hospital ETI/RSI may contribute to the component’s perceived lower quality while additional factors may include the lack in the variety of clinical cases and patient types, or only observing ETI/RSI during clinical practice placements [34].

#### Internship programme

Although no known legislation requiring newly qualified ECPs to participate in an internship programme exists, some South African EMS organisations have such programmes in place, the features of which are unknown. As part of continuous learning to prevent skill decay and promote safe and effective skill delivery, the literature recommends supervised practice for non-physicians and physicians, especially in terms of advanced airway management or RSI [12, 13, 28, 35]. Recent evidence suggests that, although newly qualified paramedics were found to be competent, there was a reported lack of organisational and health systems knowledge, leadership, clinical judgement and decision-making skills [36]. We support the development and implementation of a structured internship programme for newly qualified ECPs in SA aimed at nourishing practitioner growth to deliver safe and effective RSI, as recommended by Moodley [31].

#### Continuing professional development for ECPs

In SA, ECPs are responsible to maintain and update their knowledge and skills post-qualification [16]. The HPCSA has a Continuing Professional Development (CPD) programme requiring practitioners to accumulate Continuing Education Units (CEUs); however, the contents are not specified [37]. In the context of fast-developing airway management approaches, research sources indicate that Continuing Medical Education (CME), which include theoretical, simulated and clinical practice activities, are crucial to prevent skill decay and perform RSI safely and effectively [13]. As mentioned by the participants, short courses tailored to provide continuous advanced airway management and RSI education and training in SA exist; however, ECPs are not necessarily required to attend these or similar courses. Attendance may be influenced by practitioner preference, course availability, affordability and/or specific employee currency certification requirements. We, therefore, recommend that RSI and/or advanced airway management-specific CME activities become mandatory and form part of the accumulation of CEUs towards the existing CPD programme to ensure ECPs are up to date with current best practice.

### System requirements

#### Equipment and medication availability

We found most mandatory airway management equipment and medications to be available to ECPs, with minimal reporting of equipment sharing.

Although a small percentage of participants reported not having either a stylet or a bougie available, it should be regarded as a concern, as this is one of the fundamental airway equipment when performing ETI/RSI, recommended by local and international guidelines [13, 38]. Not using these adjuncts may affect first-pass and overall success rates and possibly increase the risk of adverse events [2]. EtCO_2_ capnography is an important and mandatory tool for the confirmation of ETT placement and a valuable diagnostic tool that could promote safe and quality patient care and optimal mechanical ventilation [39]; thus, a lower than expected availability was of concern. The low availability of VL could be contributed to the recent introduction of the device in the pre-hospital setting and could also be considered as an expensive modality; subsequently, other expenditures and equipment purchase priorities may limit procurement. However, EMS organisations in low-resource settings may consider VL, as it may improve RSI success rates, decrease associated adverse events and facilitate faster ETI especially in trauma patients and patients with a difficult airway [4, 40, 41] and should be supported by organisations and policymakers.

Correct inflation pressure with continuous cuff pressure monitoring has become important. Using the estimated predetermined volume of air to inflate the ETT cuff has been reported to be imprecise [42], while a recent SA study found ETT cuff pressure to be incorrect in 77% of patients on EC admission [43]. Although the complications of over- and under-inflation of ETT cuffs may not be noted in the emergency environment, both hold significant risks. To establish good practice, the use of an ETT cuff pressure manometer is recommended. The cost of commercial ETT cuff pressure manometers may contribute to the low availability; however, there is an alternative low-cost method to determine cuff pressure [44] which should be considered where commercial devices are unavailable.

#### Assistants to perform RSI

Consistent with EMSSA guidelines recommending at least one RSI assistant, our findings suggest that most ECPs have at least one; however, two is preferable [45]. Most international literature indicates and refers to a “team” that conducts pre-hospital RSI, highlighting the need for more than one competent assistant. More recent literature suggests that at least three knowledgeable and proficient team members should perform and assist during RSI [46].

Although airway management short courses are available, they primarily focus on educating and training those performing RSI. Nonetheless, these courses may provide some insight for non-ECP providers. To our knowledge, no short course specifically designed to equip non-ECPs with the necessary knowledge and skills to assist ECPs with RSI is currently offered in SA. A short course with the intent to train non-ECPs as RSI assistants may be extremely beneficial.

### Comprehensive clinical governance system

The implementation and practice of safe and effective pre-hospital RSI cannot be guaranteed without clinical governance and quality improvement systems [1, 13–15]. A good clinical governance system relies on a dynamic programme that supports and emphasises quality care, consultation, case feedback and clinical case reviews as part of independent practice and continuous learning [13, 14, 47]. The necessity of ongoing quality assurance, quality control and performance review was stipulated [1, 16]. Recording all RSI, whether attempted or performed, on a database is recommended [48], as it is an important component of clinical governance that can be used to facilitate improvement. In both hospital and pre-hospital settings, the implementation of a robust advanced airway programme, including the application of airway management algorithms, training, data collection and clinical reviews, has shown to significantly increase the first-pass success rate and RSI safety [49, 50].

## Study limitations

To maximise validity, the questionnaire was mindfully designed, using reputable resources, expert input, and a pilot study was conducted. However, survey responses may have been influenced by a variety of factors such as personal beliefs and perceptions leading to the potential for bias, including social desirability bias as pre-hospital RSI performed by paramedics is a controversial topic globally.

The nature of the data collection tool may have influenced the results as break-offs, participant fatigue and misinterpretation of questions are likely. Non-response bias may have been introduced if the respondents that participated were systematically different from those that did not or if some eligible participants were not reached. Minor sections in the survey required participants to recall information regarding their perceived quality of training, from the not so recent past; therefore, various cognitive biases, including recall bias, could have been introduced, likely affected by work experiences or changes in their perception. Furthermore, the non-probability sampling techniques could have produced a sampling bias that underrepresents certain demographic groups and individuals and restricted data analysis. In particular, the results were restricted by the limited representation of certain provinces; however, this may be representative of ECP distribution across the provinces in the country. Subsequently, the findings may not be generalisable to all EMS systems in SA.

Our study did not intend to provide a detailed description of current practice according to the components of the minimum standards but rather aimed to provide an overview of aspects that were deemed important for safe and effective pre-hospital RSI.

## Conclusion

In this descriptive cross-sectional study, we found that there is plentiful alignment between the components of the minimum standards of pre-hospital RSI and current practice as perceived by ECPs; however, there are also some areas for improvement.

We found specific areas of university education and training that require more in-depth investigation and possible improvement initiatives. The implementation of an internship programme for newly qualified ECPs could complement pre-hospital clinical RSI encounters while the lack of availability and minimal participation of RSI-specific CME activities should be addressed. The importance of capable RSI assistants should be underscored; therefore, the need for the development and implementation of an RSI-specific education and training intervention for non-ECPs should be strongly considered.

A trustworthy and purposeful clinical governance system including various aspects for example monitoring, evaluating and managing risks, an RSI database, provision of education and training initiatives, etc. are needed in all EMS organisations that provide pre-hospital RSI in SA. The operational utility of existing quality assurance, control and improvement tools in some EMS organisations should be evaluated while frequent revision of practice is needed to ensure alignment with recommendations and current best practice.

## Supplementary Information

**Additional file 1:.** Data collection tool

**Additional file 2: Table S1** Perceived quality of education components (*n*=76). **Table S2** Equipment sharing amongst ECPs by Organisation (*n*=32). **Table S3** Type of equipment shared amongst ECPs (*n*=32). **Table S4** Qualifications of RSI assistants (*n*=76). **Table S5** Non-ECP education and training methods assist during RSI (*n*=76)

## Data Availability

The datasets used and/or analysed during the current study are available from the corresponding author on reasonable request.

## Abbreviations

CEUs: Continuing Education Units
CME: Continuing Medical Education
CPD: Continuing Professional Development
CQI: Continuous Quality Improvement
ECP: Emergency Care Practitioner
EMS: Emergency Medical Services
EMSSA: Emergency Medicine Society of South Africa
EMOVA: Evidence-Based Management of Oxygenation, Ventilation and Airway
ETI: Endotracheal Intubation
ETT: Endotracheal Tube
HPCSA: Health Professions Council of South Africa
IQR: Interquartile Range
M: Median
NGO: Non-Governmental Organisation
PEC: Pre-hospital Emergency Care
QA: Quality Assurance
RSI: Rapid Sequence Intubation
