# Supplementary material for: Rapid sequence intubation: a survey of current practice in the South African pre-hospital setting
Source: Int J Emerg Med. 2021 Aug 17;14:45. doi: 10.1186/s12245-021-00368-3 (PMC8369626; doi:10.1186/s12245-021-00368-3)
Supplement: Supplementary file 1 — Additional file 1:. Data collection tool [file 12245_2021_368_MOESM1_ESM.pdf]

Rapid sequence intubation: A survey of current practice in the South African pre-hospital setting.

Research Study Information

Pre-hospital Rapid Sequence Intubation (RSI), performed by non-physician emergency care providers, remains a heavily debated topic worldwide. However, what could be agreed upon is that RSI is the golden standard of advanced airway management, if performed in a safe and effective manner.

Endotracheal intubation using the method of RSI in the South African pre-hospital environment is fairly new, considering that it was only approved by the Health Professions Council of South Africa (HPCSA) in July 2009, as part of the scope of practice for registered Emergency Care Providers (ECP's). In addition to the approval of this skill the HPCSA published several minimum standards, in November 2009 *(click to open link)*, that was intended as requirements for the implementation and subsequent practice of the skill within emergency medical services. In 2011 a Position Statement on pre-hospital RSI was published *(click to open link)* by key role players within the industry, endorsed by the Emergency Medicine Society of South Africa (EMSSA) and the Resuscitation Council of Southern Africa (RCSA). The Position Statement elaborated and provided clarification on three specific components (training, system requirements and clinical governance systems) pertaining to the already published minimum standards to support safe and effective delivery of pre-hospital RSI performed by ECP's in South Africa.

Recent literature highlighted a high adverse event rate with paramedic RSI in South Africa, which is concerning in terms of patient safety. It was further reported that the effectiveness of paramedic RSI, in terms of the ability to pass the endotracheal tube, was NOT found to be a concern.

The purpose of this research study is to collect data from registered ECP's in an aim to describe the current practices of Pre-hospital Rapid Sequence Intubation in South Africa in terms of the minimum standards as laid down by the HPCSA. It is important to understand the environment, conditions and the system in which RSI is taking place and how it could possibly affect the safety of RSI. Therefore, this research study aims to describe the system, and NOT the individual.

Please note that due to the nature of the research study, the survey is exclusively aimed at registered Emergency Care Practitioner (ECP) that holds a degree in Emergency Medical Care (e.g. B.Tech: EMC/BEMC/BHSc: EMC) and that are operationally active (full time, part time, voluntary) within the pre-hospital setting in South Africa.

The research study was approved by the University of Cape Town Human Research Ethics Committee (HREC) and comply with the ethical principles of the Declaration of Helsinki. For further information pertaining to the ethical considerations of this research study, the HREC can be contacted +27 21 650 3002.

This research study is in partial fulfilment of the degree M.Phil in Emergency Medicine at the University of Cape Town.

Thank you for your time and interest to participate in this research study and survey. Please feel free to contact me if you have any questions, jcbotha.vinkie@gmail.com

Researcher: Ms J.C. Botha
Supervisor: Dr W. Stassen

Co-Supervisor: Ms A. Lourens

Please click the "OK" tab below to proceed to the consent section, that will enable you to participate in the survey.

Rapid sequence intubation: A survey of current practice in the South African pre-hospital setting.

Informed Consent

Your participation in this research study is voluntary and therefore you may choose not to participate, without any disadvantage.
If you do decide to participate, you will be afforded an opportunity to withdraw at any time.
Participation in this survey do not offer any form of individual incentives, although the disseminated findings of the research study may contribute towards improved and safe patient care.
The survey is very short and would take less than 10 minutes to complete.
Please ensure that you complete all the questions, as incomplete surveys would be deemed futile.

1. I have read the information section and understand the background and purpose of the research.
2. I am registered as an Emergency Care Practitioner (ECP) at the Health Profession Council of South Africa.
3. I practice (full time/part time/voluntary) as an Emergency Care Practitioner (ECP) in the pre-hospital setting in South Africa.
4. I voluntarily agree to participate in the research study.

* 1. If you agree to the above statements, please select the "Agree" option and then click "OK" to proceed to the survey.

If you answered "NO" to any of the above statements OR wish NOT to participate in the survey, please select the "Disagree" option below.

◯ Agree

◯ Disagree

Rapid sequence intubation: A survey of current practice in the South African pre-hospital setting.

Basic Demographic Information

The purpose of this section is to acquire basic, non-identifying demographic information of the participants.

IS ESTIMATED THAT THE SURVEY WOULD ONLY TAKE 12 MINUTES TO COMPLETE. LET'S GET STARTED! IT

Please click on the "OK" button below to proceed to the questions.

2. Indicate ALL your qualifications that you obtained in emergency care during your career *(you may select more than one option).*

- [ ] Basic Ambulance Assistant (BAA/BLS)
- [ ] Ambulance Emergency Assistant (ANA/ILS)
- [ ] Critical Care Assistant (CCA/ANT)
- [ ] Emergency Care Technician (ECT)
- [ ] National Diploma in Emergency Medical Care (ANT)
- [ ] B.Tech/BEMC/BHSc:EMC (ECP)

3. Indicate the first ever YEAR of registration as an emergency care provider (e.g. BAA, ANT etc.) with the HPCSA *(if ECP registration is the first/only registration with the HPCSA, enter as such)*.

4. Indicate the YEAR of registration as an independent ECP (B.Tech/BEMC/BHSc:EMC) with the HPCSA

5. In which province of South Africa do you predominantly practice in as an ECP?

- ( ) Eastern Cape
- ( ) Free State
- ( ) Gauteng
- ( ) KwaZulu-Natal
- ( ) Limpopo
- ( ) Mpumalanga
- ( ) Northern Cape
- ( ) North West
- ( ) Western Cape

6. How would you best describe your job title within the organisation that you are employed in?

- ◯ Paramedic (ECP) - operational
- ◯ Flight Paramedic (ECP) - operational
- ◯ Lecturer/Instructor/Trainer in Emergency Medical Care/Emergency Medicine
- ◯ Manager/Administrative/Researcher in Emergency Medical Care/Emergency Medicine
- ◯ NOT permanently employed within EMS/EMC/EM (please specify)

7. When you do work operationally as an ECP (permanently/voluntary/part time), which of the following best describe the organisation/emergency medical service that you predominantly practice in?

- ◯ Public (Government)
- ◯ Private
- ◯ University/Training Institution
- ◯ Non-Governmental Organisation (NGO)
- ◯ Other (please specify)

8. How would you best describe the predominant work environment when you are operational as an ECP?

- ◯ Ground - Ambulance
- ◯ Ground - Response Vehicle
- ◯ Ground - Specialised Ambulance (e.g. ICU/Paediatric/Neonatal Transfer Unit)
- ◯ Special Events (e.g. sport standby, concerts, festivals, movie sets)
- ◯ Aeromedical - Rotor Wing
- ◯ Aeromedical - Fixed Wing
- ◯ Other (please specify)

Training

The purpose of this section is to acquire some information with regards to the training/education that you received on a tertiary level at and subsequent continuous professional development activities.

THIS IS A SHORT SECTION! - ONLY EIGHT QUICK QUESTIONS!

Please click on the "OK" button below to proceed to the questions.

9. How would you describe the OVERALL **theoretical component of the RSI** education that you received at university level *(e.g. airway anatomy, airway equipment, advance airway algorithms, the steps of RSI, pertinent pathophysiology etc.).*

Poor	Fair	Average	Good	Excellent
☆	☆	☆	☆	☆

10. How would you describe the OVERALL **simulated practical component** of the RSI education that you received at a university level *(e.g. OSCE's and Patient Simulation)?*

Poor	Fair	Average	Good	Excellent
☆	☆	☆	☆	☆

11. How would you describe the OVERALL **clinical practice component** of the RSI education that you received at a university level *(e.g. Clinical Placement/Operational Shifts/Emergency Department/Theater etc.)*?

Poor	Fair	Average	Good	Excellent
☆	☆	☆	☆	☆

12. How would you describe the **pharmacology component** of the RSI education that you received at a university level?

Poor	Fair	Average	Good	Excellent
☆	☆	☆	☆	☆

13. How would you describe the **mechanical ventilation component** of the education that you received at a university level?

Poor	Fair	Average	Good	Excellent
☆	☆	☆	☆	☆

14. How would you describe the **special circumstances component** (e.g. obese patients, traumatic brain injury patients, respiratory disorders etc.) pertaining to RSI education that you received at a university level?

Poor	Fair	Average	Good	Excellent
☆	☆	☆	☆	☆

15. After you obtained your degree, indicate the duration of the **internship programme** (worked with a qualified ECP) that you participated in, before commencing duties as an independent practitioner?

- ◯ Zero - I did not participate in any for of an internship programme (worked with a qualified ECP)
- ◯ < 1 month
- ◯ 1 - 3 months
- ◯ >3 months

16. Since you qualified/graduated from university, did you participate in any **formal** (e.g. RSI specific short course/workshop/continuous professional development activity) education/training pertaining to RSI?

- ◯ Yes
- ◯ No

If you answered "Yes", briefly provide some detail (e.g. name of the course, training provider, approved by the HPCSA, type of activity)

System Requirements

The purpose of this section is to acquire some information with regards to adequate equipment & personnel to enable you to attempt safe and effective RSI.

YOU HAVE PASSED THE HALFWAY POINT IN THE SURVEY! - ALMOST DONE! ONLY 2 SECTIONS TO GO!

Please click on the "OK" button below to proceed to the questions.

17. Which of the following airway equipment/adjuncts are available to you EVERY time that you attempt RSI?

- [ ] Suction Unit
- [ ] Oroharyngeal Airway
- [ ] Nasopharyngeal Airway
- [ ] Bag Valve Mask Resevoir
- [ ] Medical Oxygen
- [ ] Endotracheal Tubes
- [ ] Laryngeal Mask Airway
- [ ] Combitube
- [ ] King Laryngeal Tube
- [ ] Needle Cricothyroidotomy
- [ ] Surgical Cricothyroidotomy/Quicktrac
- [ ] Stylet
- [ ] Bougie
- [ ] Laryngoscope
- [ ] Video Laryngoscope
- [ ] Capnometer (EtCO2)
- [ ] Capnograph (EtCO2)
- [ ] Other Confirmation Devices (e.g. Esophageal Intubation Detector/Colorimetric Carbon Dioxide Detector)
- [ ] Electrocardiograph (ECG) with defibrillator
- [ ] Saturation Monitor (SpO2)
- [ ] Automated Oscillometric Device/NIBP
- [ ] Mechanical Ventilator
- [ ] Endotracheal Tube Cuff Pressure Manometer
- [ ] Electronic Infusion Device
- [ ] Gravitational Infusion Device/Dial-a-flow
- [ ] Heat Moister Exchanger (HME) filter
- [ ] Gastric Tubes (Orogastric/Nasogastric Tubes)
- [ ] Commercial Endotracheal Tube Ties (e.g. Thomas tube holder)
- [ ] Other equipment to perform RSI (please specify)

18. Are there any of the following equipment that is shared with another ECP unit(s) and/or kept at the base?

- [ ] NONE
- [ ] Electrocardiograph (ECG)
- [ ] Mechanical Ventilator
- [ ] Capnograph/meter (EtCO2)
- [ ] Other (please specify)
- [ ] Automated Oscillometric Device and/or Non-Invasive Blood Pressure Monitoring Device
- [ ] Electronic Infusion Device
- [ ] Video Laryngoscope

19. Which of the following medication are available to you every time that you attempt RSI and post intubation management?

- [ ] Etomidate
- [ ] Ketamine
- [ ] Midazolam
- [ ] Suxamethonium
- [ ] Rocuronium
- [ ] Vecuronium
- [ ] Morphine
- [ ] Fentanyl

Other (please specify)

20. Indicate the average number of dedicated assistant(s) that you would have available to assist you when you attempt RSI.

- ( ) 0
- ( ) 1
- ( ) 2
- ( ) 3
- ( ) >3

21. Indicate the qualification of the assistant that predominantly assist you when you attempt RSI.

- ( ) BAA/BLS
- ( ) AEA/ILS
- ( ) Emergency Care Technician (ECT)
- ( ) Paramedic (CCA)
- ( ) Paramedic (N.Dip)
- ( ) Emergency Care Practitioner (ECP)

22. Complete the following statement:*"The knowledge and skills of emergency care providers (non-ECP's) that assist me during every attempted RSI are __________________________."*

- ◯ A very high level
- ◯ A high level
- ◯ An average level
- ◯ A low level
- ◯ A very low level

23. Indicate your opinion pertaining to the following statement: *"When I attempt RSI, there is at least ONE team member that I know and have worked with previously when I performed the skill of RSI"*

- ◯ Always
- ◯ Sometimes
- ◯ Never

24. Do you know of any short courses that are available for **emergency care providers (non-ECP's)** that would provide them with the necessary knowledge and skills to adequately assist an ECP during an attempted RSI?

- ◯ Yes
- ◯ No

If you answered "Yes", briefly provide some detail (e.g. name of the course, training provider, approved by the HPCSA, type of activity)

[ ]

25. To the best of your knowledge, how did **emergency care providers (non-ECP's)** acquire the necessary knowledge and skills to assist an ECP to attempt RSI, as it is not a component of their curricula/training *(you may select more than one option, except if you select the option "I do not know").*

- ☐ **I do not know**
- ☐ RSI assistant short course/certification
- ☐ Experience/Knowledge gained from working in the Pre-hospital Setting (e.g. with an ECP)
- ☐ Experience/Knowledge gained from working in an Emergency Department (e.g. with a Physician)
- ☐ Other (please specify)
- ☐ Free open access medical education (FOAMed)
- ☐ Social Media Platforms (e.g. Facebook, YouTube)
- ☐ Medical Textbooks

[ ]

Rapid sequence intubation: A survey of current practice in the South African pre-hospital setting.

Comprehensive Clinical Governance System

The purpose of this section is to acquire some information with regards to the Comprehensive Clinical Governance System within the organisation/emergency medical service that you practice in as an ECP.

LAST EIGHT QUESTIONS TO GO!

Please click on the "OK" button below to proceed to the questions.

26. Which of the following statements best describe the clinical practice guidelines, pertaining to RSI, within the organisation that you are operationally active in? *(Please indicate the MOST correct option)*

- ◯ HPCSA Clinical Practice Guidelines
- ◯ Organisational Clinical Practice Guidelines, that are aligned with the HPCSA Clinical Practice Guidelines
- ◯ Organisational Clinical Practice Guidelines
- ◯ Autonomous practice according to provider preferred local and/or international Clinical Practice Guidelines

27. Do you know of any **formal** consultation framework (e.g. consultation with a Senior ECP, Peer and/or a Physician) within the organisation that you are working for?

- ☐ Yes
- ☐ No

28. Do you occasionally consult **informally** with a Senior ECP, Peer and/or Physician with regards to performing RSI?

- ◯ Yes
- ◯ No

29. Is there a continuous quality improvement/quality assurance department/representative within the organisation/emergency medical service that you work in?

- ◯ Yes
- ◯ No

If YES, please provide a brief description

30. Are you required to submit any form of patient care report forms/documentation/reports/checklists for an attempted/performed RSI?

- ◯ Always
- ◯ Sometimes
- ◯ Never

31. Do you receive any feedback from a continuous quality improvement/quality assurance department/representative with regards to EVERY attempted/performed RSI?

- ◯ Always
- ◯ Sometimes
- ◯ Never
- ◯ **Not applicable, there is NO CQI/QA department/representative within the organisation**

32. Are there clinical review/mortality & morbidity meetings within the organisation that discuss cases pertaining to attempted/performed RSI?

- ◯ Always (every month)
- ◯ Usually (every 3 months)
- ◯ Sometimes (every 6 months)
- ◯ Rarely (once a year)
- ◯ Never (zero per year)

33. Is there a RSI database within the organisation, that all attempted/performed RSI cases are captured?

- ◯ Yes
- ◯ No
- ◯ Unsure

Thank you for your time and participation, your contribution is appreciated.

This research study uses a non-probability snowball sampling or chain-referral sampling technique.

Please share the link (https://www.surveymonkey.com/r/QTZ6W5J) and/or QR (see below) of the survey with all other eligible individuals (ECPs in South Africa) that you know.

"Every system is perfectly designed to get the results it gets" - IHI

Thank you once again for YOUR participation in this research study!

QR Code
