# Supplementary material for: Rapid sequence intubation: a survey of current practice in the South African pre-hospital setting
Source: Int J Emerg Med. 2021 Aug 17;14:45. doi: 10.1186/s12245-021-00368-3 (PMC8369626; doi:10.1186/s12245-021-00368-3)
Supplement: Supplementary file 2 — Additional file 2: Table S1 Perceived quality of education components (n=76). Table S2 Equipment sharing amongst ECPs by Organisation (n=32). Table S3 Type of equipment shared amongst ECPs (n=32). Table S4 Qualifications of RSI assistants (n=76). Table S5 Non-ECP education and training methods assist during RSI (n=76) [file 12245_2021_368_MOESM2_ESM.pdf]

Additional file 2

Table S1 Perceived quality of education components (n=76)

Education components (combined results: average, good and excellent)	n (%)
Theoretical [a]	72 (94.7%)
Pharmacology [b]	70 (92.1%)
Simulated practice [c]	69 (90.8%)
Mechanical ventilation [d]	64 (84.2%)
Special circumstances [e]	63 (82.9%)
Clinical practice [f]	63 (82.9%)

[a] All theoretical aspects of RSI, [b] Theoretical and practical aspects of RSI medication and administration, [c] Practical classroom learning using various levels of fidelity simulation, [d] Theoretical and practical aspects of mechanical ventilation, [e] Unusual patient conditions to perform RSI, e.g., Raised Intracranial Pressure, Acute Pulmonary Oedema, Asthma, Haemodynamically Unstable Patient, Patient population, etc., [f] Supervised practice in a hospital or pre-hospital environment

Table S2 Equipment sharing amongst ECPs by Organisation (n=32)

Type of EMS organisation	n (%)
Private	17 (22.4%)
Public (Government)	12 (15.8%)
Non-Governmental Organisations (NGO)	2 (2.6%)
University/Training Institution	2 (2.6%)

Table S3 Type of equipment shared amongst ECPs (n=32)

Type of equipment	n (%)
Mechanical ventilator	30 (93.8%)
Electrocardiograph (ECG)	27 (84.3%)
$EtCO_2$ [a]	26 (81.3%)
Non-invasive blood pressure monitor	20 (62.5%)
Electronic infusion device	20 (62.5%)
Video laryngoscope	4 (12.5%)

[a]End Tidal Carbon Dioxide (CO_2)

Table S4 Qualifications of RSI assistants (n=76)

Assistant qualifications	n (%)
BAA [a]/BLS [b]	12 (15.0%)
AEA [c]/ILS [d]	40 (52.6%)
ECT [e]	6 (7.9%)
CCA [f]	5 (6.6%)
ECP [g]	4 (5.3%)
EMC [h] students	9 (11.8)

[a] Basic Ambulance Assistant, [b] Basic Life Support, [c] Ambulance Emergency Assistant, [d] Intermediate Life Support, [e] Emergency Care Technician, [f] Critical Care Assistant, [g] Emergency Care Practitioner, [h] Emergency Medical Care.

Table S5 Non-ECP education and training methods assist during RSI (n=76)

Method of education and training	n (%)
RSI assistant short course	4 (5.3%)
Working in the pre-hospital setting with an ECP	60 (78.9%)
Working in an EC [a] with a Physician	13 (17.1%)
FOAMed [b]	9 (11.8%)
Social media platforms	12 (15.8%)
Medical textbooks	11 (14.5%)
Other [c]	8 (10.5%)
Don't know	13 (17.3%)

[a] Emergency Centre, [b] Free open access medical education, [c] Other described as informal training, the overall theme was working with an ECP or taught by the ECP
